# Association between functional antibody against Group B Streptococcus and maternal and infant colonization in a Gambian cohort

**DOI:** 10.1016/j.vaccine.2017.04.013

**Published:** 2017-05-19

**Authors:** Kirsty Le Doare, Amadou Faal, Mustapha Jaiteh, Francess Sarfo, Stephen Taylor, Fiona Warburton, Holly Humphries, Jessica Birt, Sheikh Jarju, Saffiatou Darboe, Edward Clarke, Martin Antonio, Ebenezer Foster-Nyarko, Paul T. Heath, Andrew Gorringe, Beate Kampmann

**Affiliations:** a Centre for International Child Health, Imperial College London, Norfolk Place, London W2 1PG, UK,; b Paediatric Infectious Diseases Research Group, St. George’s University of London, Cranmer Terrace, London SW17 0TE, UK; c Vaccines & Immunity Theme, MRC Unit The Gambia, Atlantic Road, Fajara, Gambia; d Public Health England, Porton Down, Salisbury SP4 0JG, UK; e Statistics Unit, National Infection Service, Public Health England, 61 Colindale Avenue, London NW9 5EQ, UK

**Keywords:** Neonatal, Group B Streptococcus, Meningitis, Vaccines

## Abstract

**Background:**

Vertical transmission of Group B Streptococcus (GBS) is a prerequisite for early-onset disease and a consequence of maternal GBS colonization. Disease protection is associated with maternally-derived anti-GBS antibody. Using a novel antibody-mediated C3b/iC3b deposition flow cytometry assay which correlates with opsonic killing we developed a model to assess the impact of maternally-derived functional anti-GBS antibody on infant GBS colonization from birth to day 60–89 of life.

**Methods:**

Rectovaginal swabs and cord blood (birth) and infant nasopharyngeal/rectal swabs (birth, day 6 and day 60–89) were obtained from 750 mother/infant pairs. Antibody-mediated C3b/iC3b deposition with cord and infant sera was measured by flow cytometry.

**Results:**

We established that as maternally-derived anti-GBS functional antibody increases, infant colonization decreases at birth and up to three months of life, the critical time window for the development of GBS disease. Further, we observed a serotype (ST)-dependent threshold above which no infant was colonized at birth. Functional antibody above the upper 95th confidence interval for the geometric mean concentration was associated with absence of infant GBS colonization at birth for STII (p < 0.001), STIII (p = 0.01) and STV (p < 0.001). Increased functional antibody was also associated with clearance of GBS between birth and day 60–89.

**Conclusions:**

Higher concentrations of maternally-derived antibody-mediated complement deposition are associated with a decreased risk of GBS colonization in infants up to day 60–89 of life. Our findings are of relevance to establish thresholds for protection following vaccination of pregnant women with future GBS vaccines.

## Highlights

• As maternally-derived anti-GBS antibody increases infant colonization risk decreases.
• There is a serotype-specific threshold above which an infant is uncolonised with GBS.
• Higher anti-GBS antibody is associated with infant clearance of GBS between birth and 3 months.

## 1 Background

Group B Streptococcus (GBS) is a major cause of septicemia and meningitis in infants, affecting 1–2 in every thousand births [1]. An effective vaccine given to pregnant women would be the most powerful tool to prevent this condition globally, as it would provide protection for mothers and infants. However, field trials enrolling thousands of pregnant women and infants would be needed to assess the efficacy of such a vaccine if invasive disease was the clinical endpoint required to prove efficacy and enable licensure [2].

An alternative approach is to facilitate the development and introduction of new vaccines by identifying biomarkers that correlate with protection against disease, as were established for *Haemophilus influenzae* type B [3] and meningococcal diseases [4]. For GBS, it has been shown that high titers of naturally occurring serotype-specific maternal antibody to capsular polysaccharide (CPS) correlates with a reduced risk of neonatal disease [5]. Functional antibody assessed by OPkA appears to correlate more closely with GBS colonization in pregnant women than antibody concentration assessed by Luminex-binding assay [6]. Antibody-mediated bacterial killing has also been shown to protect infants from GBS disease and may be a more useful marker of longevity than quantifying antibodies using ELISA [5].

Since there are no validated antibody levels associated with protection from colonization or GBS disease, we assessed the relationship between antibody-mediated C3b/iC3b deposition against GBS serotypes Ia, II, III and V and an infant’s risk of acquisition of the homologous GBS serotypes.

## 2 Methods

### 2.1 Study design and participants

We undertook a prospective longitudinal cohort study in two government health centers offering antenatal care to women in the Fajara area of coastal Gambia, a low-income country with an annual birth rate of 43.1/1000 population, neonatal sepsis rate of 4.4/1000 live births [7] and neonatal mortality rate of 28/1000 live births [8]. The study was approved by the joint Gambian Government/Medical Research Council Research Ethics Committee, SCC 1350 V4. Eligibility and recruitment details have been previously described [9].

### 2.2 Study measures

Participants were followed up daily at home for 6 days and then asked to return to clinic when the infant was 60–89 days old for final follow up and vaccinations. Rectovaginal swabs were taken from enrolled women presenting in labor and cord blood was taken after delivery but prior to separation of the placenta. Nasopharyngeal and rectal swabs were taken from all eligible infants at four hours. Nasopharyngeal and rectal swabs were also taken from infants at day 6 of life and again at 60 to 89 days of life together with an infant serum sample.

### 2.3 Definitions

An infant was deemed to be colonized if either rectal or nasopharyngeal swabs were positive for GBS (or both). Colonization at or after day 6 was defined as rectal colonization as GBS is unlikely to remain a true colonizer of the nasopharynx. Persistent colonization was defined as swab-culture positive for GBS at all three time points; intermittent colonization was defined as swab-culture positive for GBS on two occasions at either birth and day 6 or birth and day 60–89 or day 6 and day 60–89, one time point is defined as swab culture positive for GBS at either birth or day 6 or day 60–89. The sites and number of infants colonized have been reported elsewhere [9].

### 2.4 Laboratory procedures

All swabs were collected in skim-milk tryptone glucose glycerol (STGG) transport medium, stored at 4 °C and transported to the Medical Research Council laboratories, The Gambia within 4 hours of collection. On arrival the samples were vortexed briefly and immediately frozen at −70 °C until processing.

All swab specimens were then inoculated into Todd-Hewitt broth supplemented with colistin and naladixic acid and were processed for isolation of GBS using standard laboratory procedures [10]. Presumptive positive GBS samples were identified by latex agglutination (Oxoid). All swabs were subjected to real-time polymerase chain reaction (PCR) [11]. All GBS positive isolates were then serotyped using conventional PCR and gel agarose electrophoresis [12].

#### 2.4.1 Complement deposition assay (CDA) [13]

Antibody-mediated C3b/iC3b deposition onto the surface of formaldehyde-fixed GBS was measured using a flow cytometric assay performed in 96-well microtitre plates [14]. Briefly, 35 μL serotype Ia, II, III or V GBS bacteria at 5 · 14 × 10^7^ CFU/mL in blocking buffer (1% BSA in PBS) were added to 10 μL IgG-depleted human plasma as the complement source [15] and 5 μL of each test serum. Plates were incubated for 7.5 min at 37 °C with shaking (900 rpm), and the bacteria pelleted by centrifugation at 3000*g* for 5 min. Supernatant was removed and the bacteria washed once with 200 μL blocking buffer. Bacteria were resuspended in 200 μL blocking buffer containing 1:500 sheep anti-human C3c FITC (Abcam) and incubated for 20 min before washing and analysis by flow cytometry.

#### 2.4.2 Opsonophagocytosis killing assay (OPkA)

The CDA values were compared to OPkA titres obtained for serotypes Ia, III and V using the HL-60 cell line as described [16], using strains 515 02/2012 (Ia), COH1 11/2013 (III) and CJBIII 03/2009 (V) (provided by Prof. Carol Baker, Baylor College of Medicine, Houston, USA). Briefly, heat-inactivated serum sample (12.5 μL) was mixed with 25 µl of bacteria revived from frozen stock (thawed and initially resuspended at 5 × 10^5^ CFU/mL in HBSS and then diluted 1/2 in HBSS containing 10% baby rabbit complement to give 6250 CFU per well), of GBS strains, 75 μL HL60 cells at a concentration of 2.6 × 10^7^ cells/mL and 12.5 µl IgG-depleted human complement [15]. Samples were incubated at 37 °C for 1 hour with shaking at 600 rpm in a Thermomixer (Eppendorf, Germany). Two positive controls and six negative controls were added to each plate. The following negative controls were used: two wells with bacteria, complement and phagocytes but without human serum; two wells with bacteria, positive control serum and complement but without phagocytes and two wells with bacteria, serum, phagocytes and heat inactivated complement. Before and after the one-hour incubation (T_0_ and T_60_), each reaction was diluted in sterile water to 1:20, 1:100 and 1:200. 10 μL of each dilution was subsequently plated by the tilt method onto blood agar plates and incubated overnight at 37 °C in 5% CO_2_. The opsonophagocytic activity was determined as the mean log_10_ reduction in GBS CFU/mL after 60 min of incubation at 37 °C compared to T_0_ (LogT_0_-LogT_60_). The lowest serum dilution analyzed was 1:30. Thus for statistical analysis, samples below the limit of detection were assigned an arbitrary titer of 15.

For both the CDA and the OPkA, results were calibrated with standard serotype-specific monovalent vaccinee serum kindly provided by Prof. Carol Baker (as above). CDA results were expressed at geometric mean (GM) of the fluorescence intensity minus the complement-only control (FI-C′).

### 2.5 Statistical analysis

The sample size was calculated on the basis of the previously observed 24% colonization rate [17], to provide at least 180 colonized women (95% confidence interval (CI) 150–202 women) and 90 colonized infants (95% CI 72–107 infants). The sample size of 180 colonized women was chosen to ensure at least 10 samples of the least prevalent serotype based on historical data from The Gambia (serotype III, 6%) [17], in order to allow longitudinal colonization analyses.

Statistical analyses were completed using STATA version 14 (StataCorp 2014, Texas) and GraphPad Prism version 6·0 (GraphPad Software Inc, La Jolla, California). The operating characteristics of the CDA were assessed as a ‘test’ for correctly classifying the paired OPkA titer as <30 (<50% killing) or ≥30 (50% or greater killing observed). For each pairwise set of comparisons, the sensitivity, specificity, positive predictive value (PPV), negative predictive value (NPV) and positive and negative likelihood ratios (LR+ = sensitivity/1-specificity and LR− = 1-sensitivity/specificity) of the CDA were calculated.

Potential differences in antibody-mediated C3b/iC3b deposition between sera from colonized and non-colonized mothers and infants were evaluated by one-way analysis of variance (ANOVA) after log transformation of data. Four groups were compared (mother colonized/infant non-colonized; mother colonized/infant colonized; mother non-colonized/infant colonized and neither mother nor infant colonized). The correlation between bacterial concentration and anti-GBS serotype-specific antibody was evaluated using Deming linear regression and 95% confidence intervals (GraphPad Software Inc, La Jolla, California). For all comparisons, p < 0.05 was considered to be significant.

### 2.6 Role of the funding source

The funders had no role in study design, data collection, data analysis, data interpretation, or writing of the report. All authors had full access to all data and the corresponding authors had final responsibility for the decision to submit for publication.

## 3 Results

750 mother/infant pairs were recruited between 1st January 2014 and 31st December 2014 [9]. Briefly, two hundred and fifty-three women were GBS-colonized at delivery (33.7%) and 186 newborns (24.8%) were colonized of whom 146 (78.5%) had GBS in both the nasopharynx and rectum. 181 newborns (24.1%) were colonized on day 6–9 and 94 infants (13.6%) at day 60–89. 525 serum samples from cord blood and 273 serum samples from infants at day 60–89 had detectable antibody-mediated C3b/iC3b deposition against any GBS serotype.

### 3.1 Correlation between CDA and OPkA

**Fig. 1:**
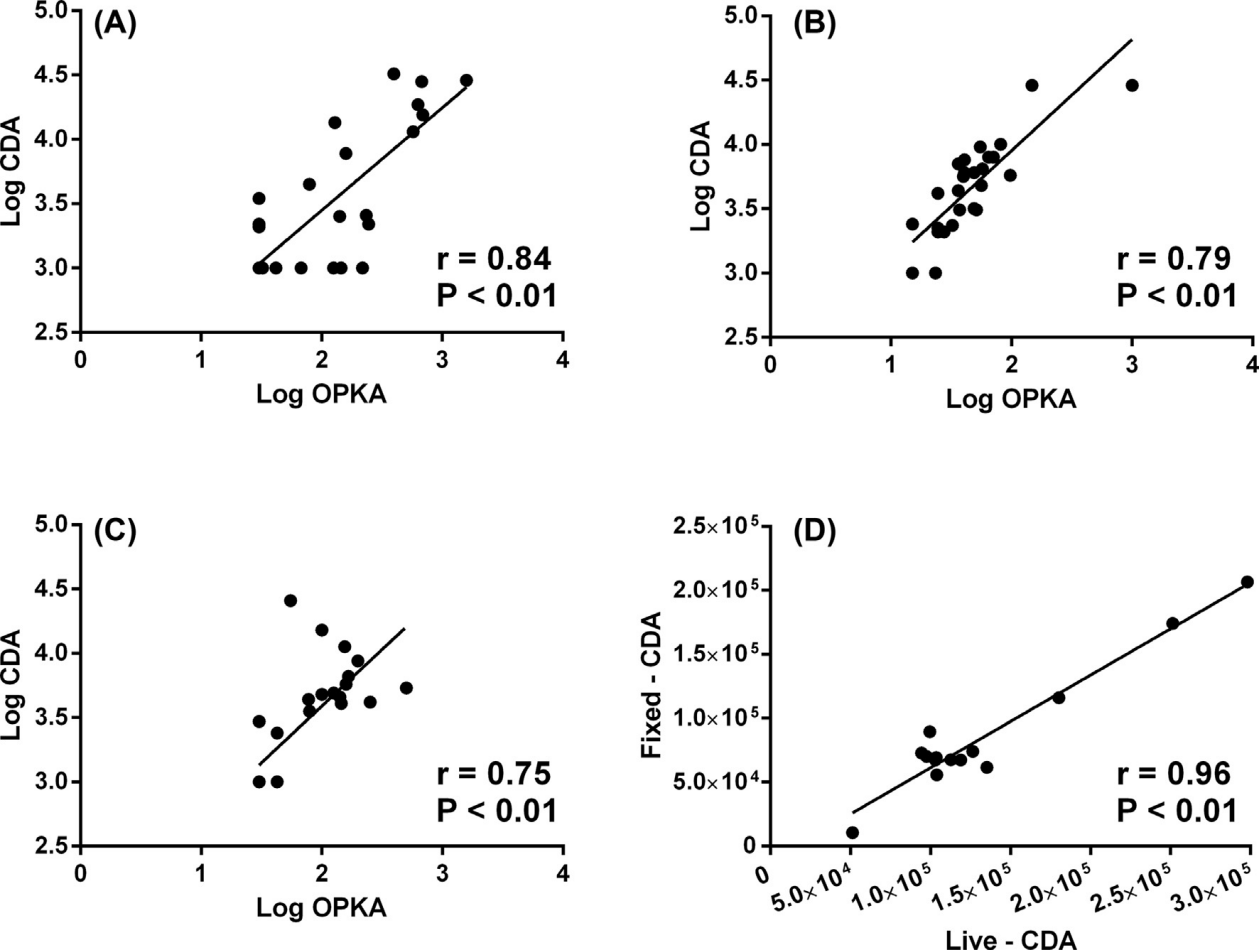
Correlation between antibody-mediated complement deposition (FI-C′) and opsonophagocytosis killing (OPkA) for serotypes Ia (A), II (B) and V (C). Panel (D) – Correlation between antibody-mediated complement deposition (FI-C′) performed with formaldehyde-fixed and live serotype III GBS. r = Pearson product-moment correlation coefficient.

The Pearson product-moment correlation coefficient (r) between the CDA and OPkA was 0.84 for STIa, 0.79 for STIII and 0.75 for STV (Fig. 1A–C). The overall sensitivity of the CDA for all serotypes was 80.7% and overall specificity was 90.0% with a weighted positive likelihood ratio of 8.07 (2.7–23.8) indicating a strong likelihood that CDA would predict opsonophagocytic killing. In addition, as formaldehyde may alter the surface components of the GBS bacteria, we compared results obtained from formaldehyde-fixed and live serotype III and V bacteria. The live and fixed assay correlation was highly significant at 0.96 (Fig. 1D).

### 3.2 Anti-GBS functional antibody in cord sera and maternal colonization at delivery

**Fig. 2:**
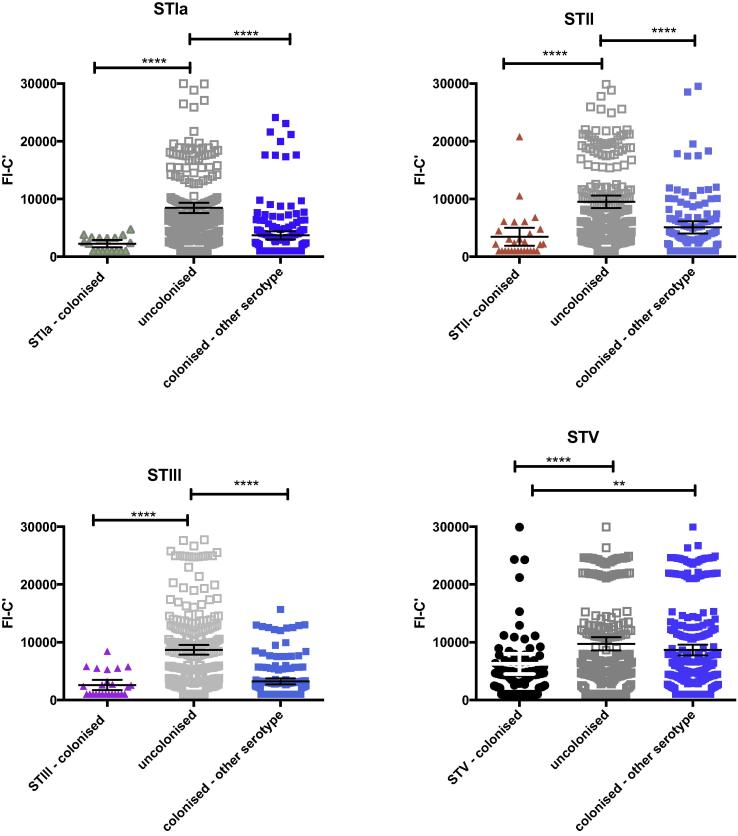
GM antibody-mediated C3b/iC3b deposition (95% CI) in cord blood onto GBS ST Ia, II, III and V. Dot plot demonstrating GM antibody-mediated C3b/iC3b deposition against GBS STIa, II, III and V in cord blood (n = 525). GM FI-C′ [95% CI] compared between mothers colonized with homologous serotype (colored shapes), non-colonized mothers (gray shapes) and mothers colonized with other serotypes (blue squares). ANOVA was calculated to compare groups *p < 0.05, **p < 0.01, ****P < 0.001. STIb not shown.

Compared to mothers who were not colonized with any GBS serotype at delivery, mothers colonized with GBS serotypes Ia, II, III or V had significantly lower GM antibody-mediated C3b/iC3b deposition in cord blood against the homologous colonizing GBS ST (Fig. 2). This remained significant after adjustment for maternal age, previous abortions, number of stillbirths, maternal anemia, maternal weight, number of antenatal clinic visits, gestation at birth and season of delivery (Supplementary Table 1).

### 3.3 Anti-GBS functional antibody in cord sera and maternal and newborn colonization dynamics at delivery

**Fig. 3:**
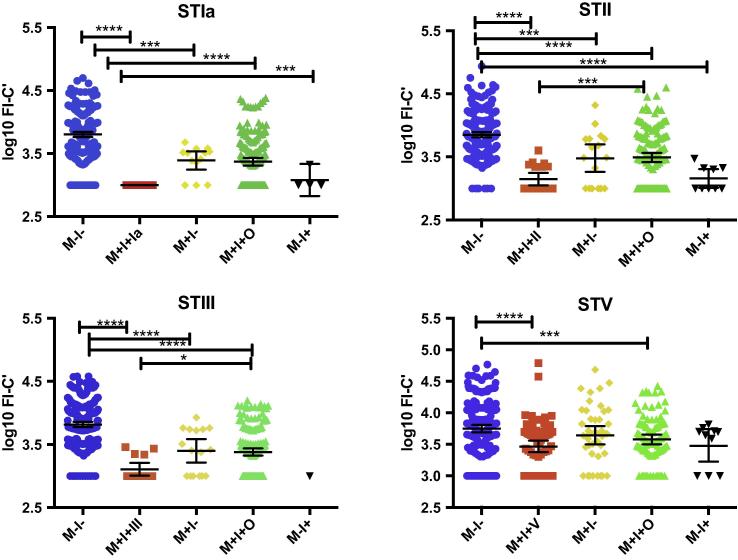
Antibody-mediated C3b/iC3b deposition comparing mother and infant colonization groups. Dot plots representing the mean and 95% confidence intervals of log_10_ FI-C′ values of antibody-mediated C3b/iC3b deposition onto the surface of whole GBS STs Ia, II, III and V bacteria (n = 525). FI-C′ – fluorescence intensity minus complement control; M−I− – neither mother nor infant colonized; M+I+(Ia/II/III/V) – both mother and infant colonized with the homologous GBS ST; M+I− – mother colonized with homologous ST, infant non-colonized with any ST; M+I+O – mother and infant colonized with different GBS ST; M−I+ – infant colonized with homologous ST, mother non-colonized with any ST. M−I− = neither mother nor infant colonized; M+I+ mother and infant colonized; M+I− = mother only colonized; M−I+ infant only colonized; ST = serotype. ANOVA was used to compare groups; *p < 0.05; ***P < 0.01; ****p < 0.001. STIb not shown.

Antibody-mediated C3b/iC3b deposition was significantly lower if mother/newborn pairs were both colonized than if mother/newborn pairs lacked colonization with the homologous GBS serotype for Ia, II, III and V (p < 0.001) (Fig. 3). If both mother and newborn were colonized with the homologous GBS serotype, the GM antibody-mediated C3b/iC3b deposition was significantly lower than if mother/newborn pairs were colonized with different GBS serotypes for II (p = 0.002) and III (p = 0.04) but not for Ia (p = 0.3) or V (p = 1.0). There was no difference between the GM antibody-mediated C3b/iC3b deposition between mother/newborn colonized pairs and those pairs where mother was non-colonized and newborn was colonized with the GBS serotype used for antibody assessment (p = 1.0 for all GBS serotypes (Fig. 3). Mother/newborn non-colonized pairs had significantly higher GM antibody-mediated C3b/iC3b deposition than pairs where infant but not the mother was colonized for STIa (p = 0.01) and STII (p < 0.001), but not for STIII or STV. In a multivariable regression analysis, higher GM antibody-mediated C3b/iC3b deposition was associated with increasing birth weight and birth during the dry-hot and wet seasons (compared to the dry cool season). Lower GM antibody-mediated C3b/iC3b deposition was associated with malaria in pregnancy and more than two tetanus immunisations in that pregnancy, when adjusted for maternal age, maternal weight, previous abortions/stillbirths, maternal anemia, number of antenatal visits, birth season and gestation (Table 1). Non-colonized mother/newborn pairs also had significantly higher GM antibody-mediated C3b/iC3b deposition onto GBS serotypes Ia (p = 0.001), II (p = 0.001), III (p < 0.001) but not V (p = 1.0), compared to colonized mothers who delivered non-colonized newborns.

**Table 1:** Multivariate linear regression model of factors associated with antibody-mediated C3b/iC3b deposition in cord blood.

Variable	Coefficient	95% CI	p-value
*Maternal factors*			
Age	0.007	−0.004 to 0.019	0.223
Weight (kg)	−0.000	−0.005 to 0.004	0.83
Parity	−0.021	−0.056 to 0.015	0.25
Malaria in pregnancy	−0.338	−0.576 to −0.099	**0.006**
Two tetanus vaccinations in pregnancy	−0.197	−0.330 to −0.064	**0.004**

*Infant factors*			
Birth weight	0.129	0.013–0.245	**0.029**
Season of birth – dry hot	0.186	0.058–0.314	**0.005**
Season of birth – Wet	0.209	0.094 to 0.323	<**0.001**
Female sex	−0.019	−0.111 to 0.073	0.678

Coefficient describes the expected change in the log odds for a log10 increase in antibody GM-FI-C′. A negative coefficient describes a reduction in log odds and a positive value describes an increase in the log odds taking into consideration all other variables in the model.

The bold values indicate values with a significance p < 0.05

### 3.4 Correlation between antibody-mediated C3b/iC3b deposition and infant colonization

**Fig. 4:**
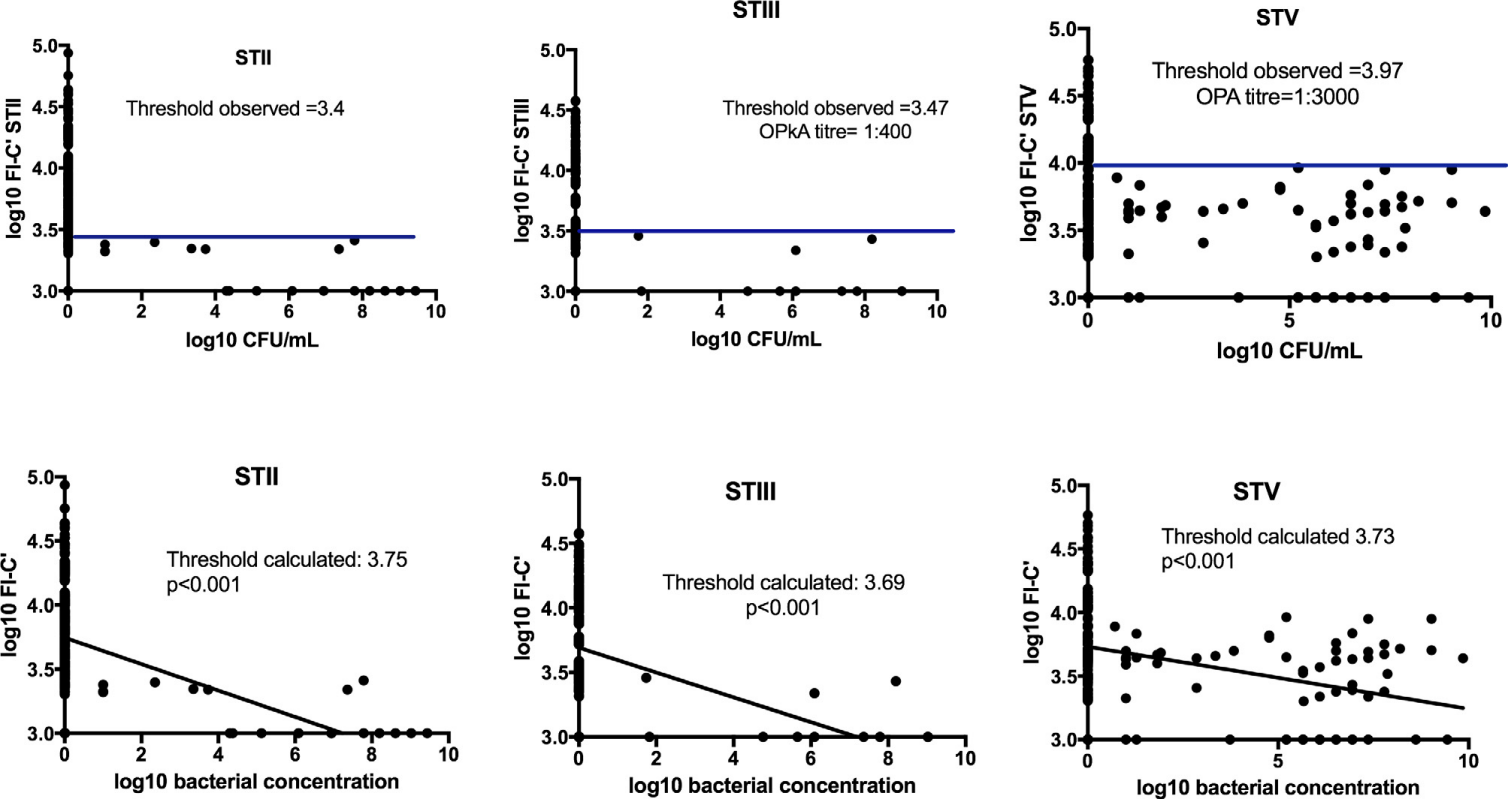
Infant GBS colonization, functional antibody threshold observed and associated Deming regression with calculated threshold. Scatter plots of log_10_GBS CFU/mL from infant swabs and log_10_ FI-C′: for GBS STII, III and V. Horizontal line represents threshold above which there was no bacterial colonization observed. STII n = 21 STIII n = 10; STV n = 62.CFU/mL – colony-forming units per milliliter; FI-C′ fluorescence intensity minus C3b/iC3b only control; ST – serotype. Deming regression of log_10_ FI-C′ against log_10_ bacterial concentration for GBS ST II, III and V comparing cord serum with CFU/mL from infant swabs at birth. Log_10_ bacterial concentration on the x-axis (CFU/mL), log_10_ FI-C′ on the y-axis.

We identified a GM antibody-mediated C3b/iC3b deposition value above which we saw no infant colonization for STII, STIII and STV. There were insufficient data to assess the relationship between anti-STIa antibody and absence of colonization (n = 3). This observed cut-off corresponded to antibody-mediated C3b/iC3b above the upper 95% CI around the GMC for STII, (log FI-C′ > 3.75, p < 0.001); STIII (log FI-C′ > 3.69, p = 0.01) and STV (logFI-C′ > 3.73, p < 0.001). (Fig. 4 and Supplementary Table 2).

### 3.5 Clearance of colonization and antibody-mediated C3b/iC3b deposition

**Fig. 5:**
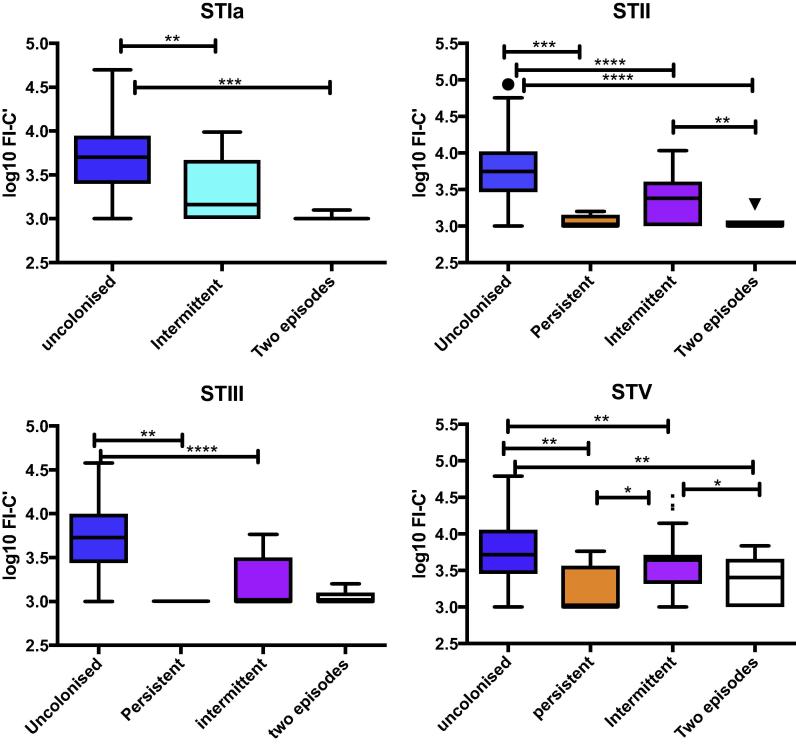
Median and standard deviation of antibody-mediated C3b/iC3b deposition in cord blood comparing non-colonized, intermittently and persistently colonized infants. Box and whisker plot with Tukey correction demonstrating median antibody-mediated C3b/iC3b deposition in non-colonized infants, intermittently colonized infants (one occasion), infants colonized on two occasions and persistently colonized infants with GBS STIa, II, III and V. FI-C′ = fluorescence intensity minus complement control, n = 525. ANOVA was calculated to determine differences between the four groups, *p < 0.05, **p < 0.03, ***p < 0.01, ****p < 0.001.

Infants who were persistently colonized with the same GBS serotype from birth to day 60–89 had significantly lower antibody-mediated C3b/iC3b deposition values than those infants who remained non-colonized with GBS STII (p < 0.01) and V (p = 0.01). No infants were persistently colonized with GBS STIa and only three infants were persistently colonized with GBS STIII. (Fig. 5). Infants who were intermittently colonized had significantly lower antibody-mediated C3b/iC3b deposition values for GBS serotypes Ia (p = 0.005), II (p < 0.001), III (p < 0.001) and V (p = 0.02) compared to infants who remained non-colonized from birth to day 60–89. Infants colonized on only one occasion had significantly higher antibody concentrations for GBS ST II (p = 0.002) and V (P = 0.05) but not for the other GBS serotypes (STIa p = 0.4, STIII p = 0.3) compared to infants colonized on two occasions. There was no significant difference in antibody-mediated complement deposition between infants colonized on two occasions and infants who were persistently colonized (GBS ST V p = 0.5, II p = 0.5) (Fig. 5).

## 4 Discussion

To our knowledge, we present the most comprehensive data of functional, maternally-derived anti-GBS antibody and infant GBS colonization from birth to 90 days of life. In particular, we demonstrate a significant negative association between antibody in cord blood and infant colonization at birth and during the first 90 days of life. The data demonstrate that compared to all other mother/infant groups, the lowest level of functional antibody is found in mother/infant pairs where both are colonized. Infants receiving the lowest concentration of functionally active antibody at birth are also less likely to clear GBS colonization between birth and day 60–89. Importantly, our data also delineate a threshold of antibody above which no infant is colonized.

We show that mothers with high antibody-mediated C3b/iC3b deposition are less likely to be colonized with GBS than mothers with lower values. This differs from the findings of a number of previous studies, which demonstrated higher anti-capsular IgG concentrations in pregnant women who were colonized with GBS than in non-colonized women at that particular timepoint [6]. It is possible that colonized women have higher anti-capsular antibody but lower functional antibody and this may account for the differences between our observations and those of others. The majority of published studies have focused on anti-capsular polysaccharide antibody concentration and have not measured antibody function. It is important to note that while ELISAs are specific for anti-polysaccharide antibodies, OPkA and complement deposition assays target both protein and polysaccharide antigens on the surface of the bacteria [16], [18]. Functional assays have been shown to better predict the duration of protection following vaccination than ELISAs [19], [20].

Only two studies have used anti-GBS opsonophagocytosis killing (OPkA) in longitudinal cohort studies of pregnant women. Baker et al. [19] used a radioactive antibody binding assay (RABA) and OPkA to GBS STIII with cord and maternal sera in 10 colonized women and found that most mothers had low OPkA titres with little variation between 28 weeks gestation and delivery. They also found that mothers colonized with GBS STIII had higher OPkA titres against GBS STIII than non-colonized mothers [19]. In contrast, a longitudinal study of 507 pregnant women by Kwatra et al. [6] found that women who were never colonized during pregnancy had higher OPkA titres in early pregnancy than those who had new acquisition of colonization during the study.

Our data show increased functional activity in non-colonized compared to colonized mothers and an inverse relationship between cord functional antibody and infant colonization. This implies that timing of acquisition of GBS by the mother is likely to be important for transfer of functional antibody from mother to child and thus protection from infant colonization. It can be postulated that if acquisition of GBS occurs during pregnancy and is not yet associated with an immune response in the mother, then the infant will be exposed to GBS at birth, receive low concentrations of functional maternal antibody and become colonized. Conversely, if a mother has previously been colonized and then cleared this colonization through the development of specific antibody then she and her infant will be non-colonized. Such infants will also be less likely to become colonized over the first 90 days of life through persistence of the maternally derived anti-GBS antibody.

To date, nearly all published work has focused on the role of antibody in protecting infants from invasive disease [5], [20], [21] with very few studies examining the relationship between maternal or infant colonization and antibody. However, this link is important because maternal colonization is a prerequisite for early onset disease and represents the major risk factor for late onset disease. Studies of *H. influenzae* type b [22]*, S. pneumoniae* [23] and *N. meningitides* [24] demonstrate that increasing antibody concentrations in serum are associated with reduced risk of colonization with these bacteria. If the same is true of GBS colonization, then increasing maternally-derived antibody through vaccination could interrupt GBS colonization and subsequent disease.

Our study has several limitations. The available data relating to correlates of protection against GBS disease have recently been reviewed [25], but there are no standardized immunoassays and the thresholds required for protection are not defined. We are thus unable to directly compare our results to other studies using different assays. As there is no standardized assay for the measurement of anticapsular antibody we were unable to measure IgG in these women. We did not use paired mother and cord sera to assess placental transfer of antibody. Instead, we used cord sera at delivery and infant sera at day 60–89 as markers of maternal antibody given that the concentration of antibody that crosses the placenta and its persistence in the infant are important for protection from disease [21], [26], [27].

Additionally, we have made some assumptions regarding infant colonization at birth. Whilst we cannot be certain that infants found to be colonized at 4 hours of birth represent true colonization rather than contamination from secretions following the birthing process, the fact that 181/186 infants remained colonized at day 6–9 indicates that this is likely to represent true colonization at this point [9].

Our CDA used a 10% complement concentration determined during assay optimization [13]. This is similar to assays for GBS developed by GSK (formerly Novartis, 10%), [16] and the Baker laboratories (14%), [28]. The maximal active complement source used in published assays is 20% [29]. In our assays, we found that 20% complement gave a high background florescence. Going forward it will be important to establish assays that use infant complement which might have different activity compared to the adult complement used in our assay.

## 5 Conclusions

In summary, our data suggests that maternal vaccination against GBS resulting in high levels of functional anti-GBS antibody is likely to lead to a reduction of maternal and infant colonization at birth and up to day 89 of life. Our results also demonstrate that it might be possible to develop an immune correlate of protection from colonization, which could subsequently be validated during vaccine trials where colonization is additionally evaluated as an endpoint.

## Declaration of interests

KLD, AF, MJ, FS, FW, HEH, ST, AG declare no conflict of interests. PTH and BK are occasional advisors to Pfizer and GSK vaccines; The MRC Unit The Gambia has previously received funding for vaccine trials, including vaccines produced by Pfizer and GSK.

## Funding

This work was supported by a Wellcome Trust Clinical Research Training Fellowship to KLD (WT2015) and the Thrasher Research Fund (BK: 12250). BK is also supported by grants from the UK MRC (MC_UP_A900/1122, MC_UP_A900/115) and the UK Medical Research Council (MRC) and the Department for international development (DFID) under the MRC/DFID Concordat arrangement.

Preliminary results have been presented at INMIS 2015, Fajara, The Gambia.

## Authors’ contributions

KLD and FW had full access to all the data in the study and take responsibility for the integrity of the data and the accuracy of the data analysis. KLD, Imperial College London and FW, Public Health England, undertook the data analysis of the study. Profs Kampmann and Gorringe contributed equally to this article.

*Study concept and design:* Le Doare, Kampmann, Gorringe, Heath.

*Acquisition of data:* Le Doare, Humphries, Gorringe.

*Analysis and interpretation of data:* Le Doare, Gorringe, Kampmann, Warburton.

*Drafting of the manuscript:* Le Doare, Heath, Gorringe, Kampmann.

*Critical revision of the manuscript for important intellectual content:* Le Doare, Antonio, Foster-Nyarko, Jarju, Clarke, Darboe, Taylor, Humphries, Gorringe, Heath, Kampmann.

*Statistical analysis:* Le Doare, Warburton.

*Obtained funding:* Le Doare, Kampmann.

*Administrative, technical, or material support:* Birt, Faal, Jaiteh, Sarfo, Taylor, Jarju, Darboe, Foster-Nyarko, Humphries, Le Doare, Kampmann.

*Study supervision:* Kampmann, Gorringe, Heath.
