# Supplementary material for: Association between functional antibody against Group B Streptococcus and maternal and infant colonization in a Gambian cohort
Source: Vaccine. 2017 May 19;35(22):2970–8. doi: 10.1016/j.vaccine.2017.04.013 (PMC5432431; doi:10.1016/j.vaccine.2017.04.013)
Supplement: Supplementary data 1 [file mmc1.docx]

**Supplementary Table 1- Multiple regression model demonstrating the reduction in antibody-mediated C3b/iC3b deposition associated with maternal colonization with homologous GBS ST compared to non-colonized mothers**

|  | STIa  FI-C’, (95% CI) | STIb  FI-C’, (95% CI) | STII  FI-C’, (95% CI) | STIII  FI-C’, (95% CI) | STV  FI-C’, (95% CI) |
| --- | --- | --- | --- | --- | --- |
| Mother colonized with homologous GBS ST | -6174  (-9273 to -3074)**** | -1924  (-6287 to 2438) | -5866  (-9106 to -2626)**** | -6731  (-9067 to -4394)**** | -3982  (-6004 to -1959)**** |
| Mother colonized with another GBS ST | -5166  (-6466 to -3866)**** | -885  (-1931 to 160) | -4871  (-6582 to -3160)**** | -5476  (-6626 to -4326)**** | -4435  (-6574 to -2290)**** |

*All GM FI-C’ figures demonstrate the reduction in GM FI-C’ compared to mother/infant group where neither was colonized. Model adjusted for maternal age, weight, anemia, gestation at delivery, previous stillbirths/spontaneous abortions, number of antenatal clinic visits and season at delivery. ****=p<0*·*0001*

Supplementary table 2 – Relationship between antibody-mediated C3b/iC3b deposition and bacterial concentration (CFU/mL) for GBS ST Ia, II, III and V

| Serotype | GMC and 95% CI FI-C’ | Intercept (95% CI) | Slope (95% CI) | P |
| --- | --- | --- | --- | --- |
| Ia | 3.59 (3.55-3.63) | 3·70 (3·64-3·75) | -0·09 (-0·15 to -0·03) | <0·001 |
| II | 3.66 (3.62-3.70) | 3·75 (3·7-3·79) | -0·1 (-0·14 to -0·07) | <0·001 |
| III | 3.59 (3.56-3.63) | 3·69 (3·65-3·74) | -0·1 (-0·14 to -0·05) | <0·01 |
| V | 3.66 (3.62-3.70) | 3·73 (3·68-3·78) | -0·05 (-0·07 to -0·03) | <0·0001 |
